# A Case of Erysipelas and Subsequent Rupture of the Stomach

**Published:** 1857-01

**Authors:** D. W. Young

**Affiliations:** Aurora, Illinois


### ART. IV.—*A Case of Erysipelas and Subsequent Rupture of the Stomach.* By D. W. Young, M. D., of Aurora, Illinois.

Enclosed, I send you the particulars of a case of erysipelas and subsequent rupture of the stomach, that occurred in my practice, which, if you deem of sufficient importance, you are at liberty to publish in your valuable and widely circulated Journal:

On the 24th day of August, 1856, I was called to visit Mrs. B., an old resident of our city, who was about 27 years of age, of bilious temperament, and who had never been sick except with an occasional attack of ague.

She informed me, on my arrival at her house, that, on the previous day, while engaged in washing her dinner dishes, and wiping a small earthen mug, the handle of which had been broken off, the thumb of her right hand was cut by a small projection that remained of the handle, and, a few hours after the accident, her thumb began to swell, and it inflamed and became most excruciatingly painful.

She applied a little salve, and passed the night as best she could; in the morning, I found her complaining bitterly of her thumb.

Upon examination, I found a small incision on the inside of her right thumb near its second joint. It was considerably swelled and inflamed, and very painful. Pulse 97 per minute, full and quick; tongue dry and coated.

She said her bowels had been costive for several days. I ordered hot poultices to the thumb and hand, and left her

℞ Hydg. sub. mur., grs. xij.
Soda bicarb., grs. vj.
Rhei turci pulv., grs. v. Misce.

And take immmediately.

August 29, 7 o'clock, A. M. I found Mrs. B. no better. Bowels had moved quite freely several times; hand and arm were swelled, and presented the appearance of rapidly spreading erysipelas. Her pulse 110, full and h rd; had a hard chill that morning, and then a high fever, with severe pain in the head; skin hot and dry.

Hot fomentations had been continued since my last visit, without any apparent benefit.

I ordered the saturated tincture of iodine to be applied freely over the hand and arm as far as the swelling and inflammation have extended; to be followed in half an hour thereafter, with constant irrigation of ice water, and left her .

℞ Quinia sulph., grs. xx.
Ipecac. pulv., grs. ij.
Papaver. pulv., grs. ij. Misce.

Div. in chart No. 4, one to be taken every two hours until all are taken.

August 30, 7 o'clock, A. M. I found Mrs. B. much worse, the inflammation and swelling had extended nearly to the elbow-joint, and now presented the appearance of a severe case of traumatic erysipelas.

The hand and arm, as far as the elbow-joint, were enormously swelled, and the skin presented a remarkably red and shining appearance and was very hot and hard.

She complained of a peculiar burning, tensive, pricking and smarting pain, and the least pressure or touch caused her pain and uneasiness.

Her pulse was 123, very full and hard; tongue dry and furred; bowels hard, moved once or twice since my last visit; urine scanty and high colored; had high fever, and some delirium during the night.

I ordered venesection largely, and the argenti nitras, in the stick, to be applied freely to the hand and arm,—to be followed with the ice water,—and left her

℞ Ant. tart., grs. iij.
Ipecac. pulv., grs. iij.
Morphia sulph., grs. ij.
Aqua fontis, ℥iv. Misce.

And give a teaspoonful every three hours.

4 o'clock, P. M. I found Mrs. B. feeling a little better, had slept some that afternoon; pulse 120, not so full nor quick; neither swelling nor inflammation had extended since morning.

Continued same prescription.

August 31st, 6 o'clock, A. M. I found Mrs. B. about the same as when I left her last evening.

She thought she had rested better that night than any night since her sickness began. Her pulse was 120, quicker, but not as full as at last visit; bowels had not moved since last visit; tongue still dry and furred; had some pain in the head, and a little fever; had no appetite.

She thought that her milk was drying up, and that her babe, which was about two months old, had better be weaned, but as the child seemed perfectly healthy, and was growing nicely, I advised her to continue nursing it. I ordered

℞ Massa hydg., grs. ij.
Ipecac. pulv., grs. iij.
Quinia sulph., grs xv.
Papaver pulvis, grs. iij. Misce.

Ft. in pill, No. 6, one pill to be given every three hours.

7 o'clock, P. M. I found Mrs. B. feeling much better; had perspired freely since taking the pills. Her bowels had moved twice during that day; tongue a little moist; pulse 119, soft and steady.

I thought there was a little more swelling about the elbow, and, therefore, applied the argenti nitras again.

Ordered a continuance of the pills.

September 1st, 7 o'clock, A. M. I found Mrs. B. feeling much worse; she had several chills during the night, and complained of deep seated pain and throbbing in the hand and arm.

There were also numerous small blisters arising over the hand, together with other symptoms of suppuration. Her pulse was 124, small and quick; bowels had not moved since my last visit; she had more fever and thirst; the swelling was about the same that it had been for several days.

I opened the blisters, and ordered hot poultices to the hand and arm, to hasten suppuration, which I was satisfied was certain to follow, and I left her

℞ Tinct. cinchona comp., ℥ij.
Tinct. gent. comp., ℥ij. Misce, and give a teaspoonful every two hours.

7 o'clock, P. M. I found Mrs. B. about the same as when I left her in the morning.

I ordered a continuance of the poulticing and tonics.

September 2d, 7 o'clock, A. M. I found Mrs. B. feeling very badly; hand and arm had pained her very much all night; felt exceedingly irritable and nervous; pulse 126, small and thready; tongue moist and cleaning; bowels regular.

I made several free incisions in the hand, which discharged freely. I ordered poultice, composed of yeast and charcoal, to the hand and arm, and left her

℞ Vinum rub., Oss.
Quina sulph., grs. xxx.
Ferri citras, ʒiij.
Elix. vitriol, gtt. xxx. Misce, and give a teaspoonful every four hours.

7 o'clock, P. M. I found Mrs. B. feeling much better. Her hand had discharged freely, and the swelling had gone down considerably.

I ordered a continuance of the same treatment.

September 3d, 9 o'clock, A. M. I found Mrs. B. improving finely; pulse 96, soft and steady; tongue clean and natural; bowels regular; appetite better.

I ordered a continuance of the same treatment.

September 5th, 10 o'clock, A. M. I found Mrs. B. bolstered up in bed nursing her babe. She said that she was nearly well, and that she now had a plenty of milk for her babe, which had grown finely during her sickness, and had not had a sign of diarrhœa or fever.

The swelling had entirely left her hand and arm, and they were healing as fast as we could reasonably expect. Her appetite was good; digestion seemed perfect; and her bowels were regular.

I advised her to continue the wine and iron.

Her improvement continued steadily and satisfactorily, until about the tenth or twelfth of September, about which time she had several abscesses form in different parts of her system. She had two or three about the neck and superior extremities: one large one on each hip. They soon suppurated and, after being lanced, discharged and healed.

Soon after the formation of these abscesses, there appeared a small pimple on the back part of her left leg, just below the knee-joint, which was quite red, and very sensitive. It lasted two or three days, and then dried up, but immediately thereafter, and directly under the place where the pimple had been, there appeared a deep and circumscribed swelling, which was most excruciatingly painful, and was about three or four inches in circumference. It did not swell so as to elevate the integument much, but immediately over the tumor the skin presented a peculiarly blue and rough appearance.

The leg became firmly flexed and fixed on the thigh, and the least attempt to extend it caused her great suffering. I applied the tincture of iodine freely, and kept it covered with hot poultices for about three or four days, after which the pain suddenly stopped, the swelling gradually subsided, and the tumor finally disappeared without suppurating; the limb became extended and assumed its natural appearance.

In about four days after the tumor subsided, her left foot began to swell, and became œdematous, remaining so about a week or ten days, when it gradually subsided, and she regained the entire use of her leg and foot.

She suffered much pain with the tumor and abscesses, and became considerably emaciated: notwithstanding, her appetite was craving, her digestion good, and her bowels regular.

September 29th, 10 o'clock, A. M. I was called to visit Mrs. B. whom I found complaining of severe pain in the stomach. Upon inquiry I learned that she had been eating freely of plums, and soon after was attacked with pain in the stomach, which I supposed was caused by indigestion. I gave her an emetic of ipecac, which operated well in a short time, and she threw up a quantity of plum skins, and other undigested food. I then gave her an anodyne, which relieved her in a few moments, and I heard nothing more from her until the sixth day of October, when I was again called to see her, and found her afflicted with an attack of ague, complicated with a sort of dysenteric diarrhœa, which she informed me had troubled her for two days. I left her

℞ Quina sulph., grs. xv.
Hydg. cum. creta., grs. x.
Papaver pulvis., grs. ij. Misce.

Div. in chart, No. 4. Take one every three hours.

October 7th, 9 o'clock, A. M. I found Mrs. B. feeling much better. The fever had entirely left her, and her bowels had not moved since she began to take the powders.

She said that she had been feeling first rate since she had recovered from the erysipelas, until she was attacked with ague, a few days before. She had gained very much in flesh since I had visited her before, and had recovered the use of her joints, except the second joint of her little finger, which was slightly flexed and anchylosed.

Notwithstanding she had several attacks of ague, and a protracted attack of traumatic erysipelas, *still she did not have the least appearance of any typhoid symptoms. I could not discover the least sign or symptom of any disease of the stomach whatever.*

October 17th, 8 o'clock, P. M. I was sent for in great haste to visit Mrs. B. On my arrival at her heuse, I found her rolling and tumbling about the bed, apparently in the greatest agony.

She was cold and clammy, and nearly pulseless. Her pupils were dilated and her countenance had a peculiar cadaveric appearance. She complained of intense pain in her stomach, and said that she had a great load at the pit of her stomach, and that she must throw it up. She said that she had tried to vomit several times, but had not thrown up anything.

I immediately inquired of her mother-in-law, who lived in another part of the same house, how she was first attacked, of whom I learned the following particulars:

She said that Mrs. B. had been improving nicely since my last visit, and on that day had apparently been feeling well; that she had been in her room at about four o'clock that afternoon with her babe, feeling very cheerful and happy. Mrs. B. told her that she was expecting company on the next day, and consequently had worked very hard in making the necessary arrangements for the entertainment of her expected guests. The Irish servant girl told me that Mrs. B. had eaten largely of some apples while engaged in paring them for pies; also, that at supper she ate some sour pickles, and then, to cap the climax, when her husband returned from his office, he brought her some nuts, of which she also ate freely.

Soon after eating the nuts, (which was about six o'clock,) she was attacked with the pain and cramps in her stomach. She said that her bowels had moved twice that day regularly. Her abdomen was somewhat bloated and slightly tympanitic. I at first diagnosticated flatulent colic arising from the multiplicity of indigestible articles of food taken into the stomach.

I immediately prepared a solution of ipecac, and gave her freely of it, but it did not have the least effect whatever. I also ordered hot fomentations to the bowels, and gave her injections to move her bowels, but she could not retain them for a moment, and so they, too, failed to have any effect. I had already given her anodynes and stimulants, but they had also had no effect.

The tympanitis had now increased so as to interfere very much with respiration; and her circulation was becoming more feeble.

Upon careful examination of the abdomen I became pretty well satisfied that the tympanitis was external to the intestines, and, therefore, began to suspect that there might be a rupture of the intestines or stomach.

I immediately informed her husband of my fears, and that I thought it was the stomach, as that had failed to act upon anything given.

At first Mrs. B. seemed very much excited, but now she had become perfectly quiet, and said that she was not at all alarmed, but wished me to relieve her bloating as soon as possible, as she could not breathe, and if not relieved must burst.

I had now fully begun to appreciate the importance of the case, and its inevitable result. I again communicated my fears to the husband, and requested council, when Dr. O. D. Howell was immediately sent for.

Mrs. B.'s abdomen had now become enormously distended, and she was constantly gasping for breath, and said that she must pass her urine. She raised up to use the vessel, passed a small quantity of urine, and then laid herself back upon her bed completely asphyxiated, and expired just as Dr. Howell arrived at the house, which was about half-past ten o'clock, and about four hours from the time that she was first attacked.

Dr. Howell, after having heard the particulars of the case, came to the same conclusion that I had: that there probably was a rupture of the intestines or stomach, and we immediately solicited the privilege of making a post-mortem examination, which request was kindly granted us, and two o'clock, P. M., of the next day, was fixed as the hour.

*Post-mortem examination sixteen hours after death.*—Present, Drs. Howell, Allaire, Eastman, Carr, Higgins and Young.

The abdomen had remained distended to its utmost capacity. As soon as the scalpel entered the abdominal cavity, in making the incisions, the gas escaped with a loud and whistling noise, at the same time giving off the peculiar odor of cider or fermented apples, a fact which all present noticed.

The abdomen immediately collapsed, and after completing the incisions we found numerous pieces of apple, pickle, and nuts, mixed among the viscera of the abdomen. We found a very large amount of adipose substance covering the abdomen, notwithstanding her extreme emaciation. The bowels were perfectly healthy and not ruptured, but when we examined the stomach, we found it ruptured to the extent of at least five or six inches, extending from its splenic end round the greater curvature, and its contents, of course, discharged into the abdominal cavity.

The mucous membrane of the stomach presented a peculiarly dark and mottled appearance. It was very much injected, thickened, and softened; but we found no signs of ulceration in it whatever. The increased development seemed to be entirely confined to the mucous follicles, which formed numerous granules, which were disseminated through the entire membrane, and gave it a sort of mammillated appearance. Upon the outer surface of the stomach we found two or three places, to the extent of from four to five inches in length, and one and two inches in width, where the peritoneal coat was entirely wanting. The edges of the peritoneal coat, at these vacant places, presented, under the magnifying glass, all the appearances of healthy granulations—a fact which most or all of the physicians present examined and acknowledged. The edges of the peritoneal coat round the vacant places, were elevated and thickened, but we examined them carefully, and thoroughly, and were satisfied that they were not rolled under.

We did not find any abnormal appearance of any of the viscera of the abdomen except the stomach.

*Query.*—Had she probably had a masked attack of sub-acute inflammation of the stomach, producing the peculiar appearance of its mucous membrane, or was this appearance caused by chemical action of the gastric juice upon the blood and mucous membrane after death? What was the probable cause of the openings in the peritoneal coat of the stomach? Was it ulceration? Is it possible to have inflammation and ulceration of a part of the peritoneum without having marked symptoms of an extensive peritonitis? Editors please answer.

